# Comparison of Transcriptome Differences in Scales of Two Closely Related Snake Species (*Lycodon rufozonatus* and *Lycodon rosozonatus*)

**DOI:** 10.3390/ani15071061

**Published:** 2025-04-06

**Authors:** Ke Sun, Anqiong Lu, Yu Xu, Fei Zhu

**Affiliations:** School of Life Sciences, Guizhou Normal University, Guiyang 550025, China; sunkeke1999@163.com (K.S.); 18485778038@163.com (A.L.)

**Keywords:** *Lycodon rufozonatus* and *Lycodon rosozonatus*, color differences, RNA-Seq, genetic variation, differential expression

## Abstract

The diversity of skin colors observed in reptiles is indicative of their adaptation to different ecological niches, with these color variations playing essential roles in survival and reproduction. The present study focused on two closely related species (*Lycodon rufozonatus* and *Lycodon rosozonatus*) within the *Lycodon* genus that exhibit the closest color and morphological similarities. We performed RNA sequencing on the scales of both species and obtained 350,346,591 and 331,537,523 clean reads, respectively. A comparative analysis of the Kyoto Encyclopedia of Genes and Genomes (KEGG) and Gene Ontology (GO) databases revealed that the scales of the two species exhibited similar patterns of gene enrichment. Nevertheless, the results of the PCA and the t-SNE analysis demonstrated notable differences between the scales in the diverse color variations observed in the two species. Concurrently, we conducted a comparative analysis of the skin color-related genes and the differentially expressed genes, which revealed three genes exhibiting notable disparities: RU_DN1145_c3_g2 (*mreg*), RU_DN10511_c0_g1 (*notch1*), and Unigene11172 (*notch1*). In light of the aforementioned results, we hypothesize that the discrepancy in the scale color between the two species is attributable to alterations in specific gene loci and variations in expression levels. The data presented herein provide a molecular basis for future studies on the genetic and habitat adaptation functions of scale color differences in reptiles.

## Simple Summary

The pigmentation of animal bodies can be influenced by various factors, including temperature, light, habitat characteristics, and the visual systems and behavioral patterns of prey and predators. The development of diverse body colors in animals has been attributed to long-term selection pressures. To explore the cellular mechanisms underlying color differences between species, two closely related snake species (*Lycodon rufozonatus* and *Lycodon rosozonatus*) were selected for study. We used transcriptome RNA sequencing to analyze the differences in the scale color in different parts of these two snakes and identified two different types of genes responsible for the color change. The results demonstrated that the mutation sites of common color genes in the two species exhibited discrepancies at various sites within seven genes. Notably, two genes, *mreg* and *notch1*, were substantially downregulated in *L. rosozonatus*. The current study suggests that color adaptation in reptiles is influenced not only by visible changes in skin pigmentation but also by underlying biological processes.

## 1. Introduction

Skin coloration is an important phenotypic trait that has multiple adaptive functions and functions in biological processes, including species identification, thermoregulation, camouflage, warning or threatening predators, social communication, and selective mating [1,2,3]. Skin color in vertebrates originates from specialized color cells. These cells are known as chromatophores [4,5,6] and include three main types: melanophores, xanthophores, and iridophores, derived from neural crest cells (NCCs) [7]. Melanophores synthesize the pigment melanin, whose color ranges from black to reddish brown. Xanthophores store red-to-yellow pigments, including pteridines synthesized by the cell and carotenoids obtained from the diet. Iridophores contain platelets of crystallized purines, which can appear blue, white, or iridescent, depending on their structure. The arrangements and interactions of these chromatophores result in different hues and colors [8], which typically indicate specific functions, such as intra-/interspecific communication, sexual selection, and adaptive survival [9].

In vertebrates, the conserved melanocortin 1 receptor (*MC1R*) and its antagonist agouti signaling protein (ASIP) form the cornerstone of melanogenesis, governing the balance between eumelanin (black/brown) and pheomelanin (red/yellow) pigments [10,11]. However, reptiles exhibit unique adaptations, uniquely exploiting structural colors through iridophores, specialized cells containing guanine nanocrystals [12]. Examples of color-producing structures include the ordered keratin matrix in bird feathers, chitin layers in butterfly wings [13], and purine crystals in the skin of fish and amphibians [8]. These structures generate a diverse range of colors, including iridescence, by selectively reflecting specific wavelengths of light [14].

Squamate reptiles (lizards and snakes) are among the most colorful vertebrates, and their diverse color patterns serve as an adaptive mechanism enabling them to thrive in a wide range of habitats [15,16]. When a species undergoes a change in body color, it is important to consider the underlying genetic factors that may influence this phenomenon. Understanding the process of generating internal and interspecies type variation and the relationship between the division of populations and the formation of species is a basic goal of evolutionary biology [17]. Within the same species, color variation is known as polymorphism. Polymorphic species are those in which multiple discrete phenotypic variants coexist within a population. Such geographic variation in polymorphism may act as a precursor to speciation if the processes generating geographic variation in the morph frequencies also promote phenotypic and genetic differentiation, leading to reproductive isolation among populations [18,19,20]. Indeed, color polymorphism has been shown to accelerate speciation in birds [19]. One such species is the Hispaniolan trunk anole (*Anolis distichus*), a widespread and common anole from Hispaniola and the Bahamas, whose dewlaps can range from entirely pale yellow to dark red. The majority of the dewlap variation observed in Hispaniolan trunk anoles is found among geographically distinct populations that have been identified as subspecies. While some of these subspecies may warrant recognition as full species, the majority appear to experience some degree of intergradation when they come into contact [21,22,23]. Similarly, polymorphisms are also observed in Asian vine snakes (*Ahaetulla prasina* and *Ahaetulla flavescens*), with individuals of these two species exhibiting distinct morphological and molecular characteristics. This has led to their classification as separate species in subsequent taxonomic revisions [24].

Previous research on skin color has focused on three main areas: morphology, biochemistry, and ecology [25,26]. However, relatively few investigations have investigated the molecular mechanisms involved in pigment synthesis in chromatophores (melanophores or xanthophores) [27,28,29]. Concurrently, research into the differential expression of specific genes regulating color in reptiles has concentrated on sexual dimorphism in lizards [28,30]. This phenomenon is also observed in snakes, with the skin color of ball pythons (*Python regius*) demonstrating variation between individuals [31]. Many of these phenotypes are reminiscent of patterning changes that have occurred within the snake lineage across evolutionary time. These include patterns that are thought to be adaptive for certain behavioral ecologies [32,33,34]. Although many polymorphic species present population-level variations in their morph composition and frequency, few studies have examined geographic variation in polymorphisms with a particular emphasis on its underlying causes and evolutionary consequences [20]. For example, the process of the morph frequency in the Australian tawny dragon lizard (*Ctenophorus decresii*) is characterized by notable variation in the throat coloration among males within and between populations [35]. Previous research on *C. decresii* has also revealed significant variations in morphology among different populations. However, this geographic variation has not yet been explicitly documented [36].

To gain deeper insight into the genetic processes that regulate the development of scale color patterns, we investigated the differences in coloration observed between two closely related species of snake (*L. rufozonatus* and *L. rosozonatus*). *L. rufozonatus* and *L. rosozonatus* are two closely related congeners of the snake family Colubridae [37]. *L. rufozonatus* is distributed mainly in China, Japan, Republic of Korea, Laos, Vietnam, Russia, and other regions, whereas *L. rosozonatus* is endemic to Hainan Province, China. The arrangements of the scale colors between the two are strikingly similar, with the former displaying black and red scales in an alternating pattern throughout the body and the latter exhibiting black and pink scales in an alternating pattern throughout the body. Consequently, we used an RNA sequencing approach to examine the gene expressions and characterize the scale transcriptomes of these species. We compared the differential gene expression between color morphs. These findings provide a foundation for further investigations into the habitat adaptation and evolutionary relationships of these two snake species.

## 2. Materials and Methods

### 2.1. Animal Collection

**Figure 1:**
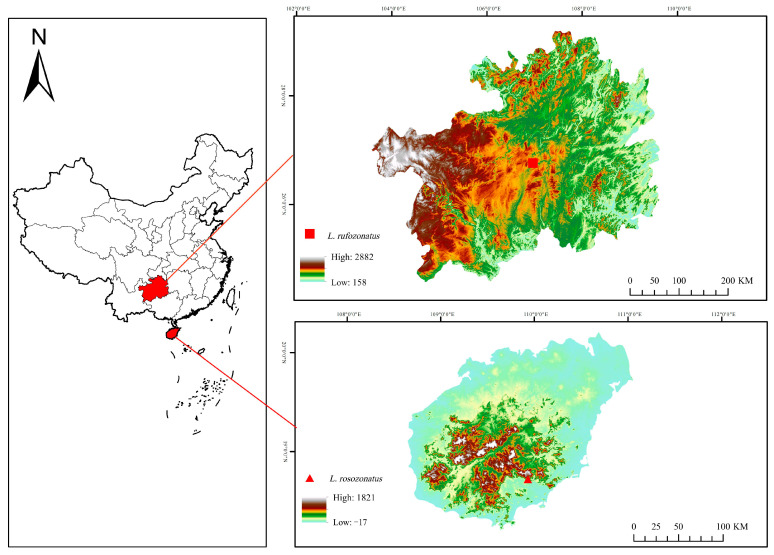
Species maps of *L. rufozonatus* and *L. rosozonatus*. The red square icon indicates the sampling site of *L. rufozonatus* in Guizhou Province, and the red triangle icon indicates the sampling site of *L. rosozonatus* in Hainan Province.

*L. rufozonatus* was collected from Guiyang city, Guizhou Province (individual numbers: GS0668, GS0707, and GS0708), whereas *L. rosozonatus* was collected from Diaoluo Mountain, Hainan Province (individual numbers: GS0676, GS0689, and GS0690) (Figure 1). All six individuals were collected from adult snakes. Once the animal collection was complete, first, the snakes were euthanized by injecting alcohol into their abdominal cavities. Subsequently, black and red scales (0.5~1 cm) from the middle parts of the bodies of three *L. rufozonatus* were excised. Likewise, pink and black scales (0.5~1 cm) from the middle parts of the bodies of three *L. rosozonatus* were collected and immediately preserved in tissue tubes containing liquid nitrogen. This resulted in the obtainment of 12 skin and scale samples. The tissues were immediately frozen in liquid nitrogen after dissection and then stored at −80 °C until RNA extraction. All the methods were carried out in accordance with relevant guidelines and regulations. Animal handling and experimental procedures were approved by the Ethics Committee of Guizhou Normal University.

### 2.2. Whole-Genome Resequencing and RNA Extraction

High-quality genomic DNA was isolated from *L. rufozonatus* and *L. rosozonatus* scale tissue with the Qiagen DNeasy Plant Mini Kit (univ-bio, Shanghai, China). MGI libraries were constructed with the MGIEasy Universal DNA Library Prep Set (BGI, Shenzhen, China). In brief, 1–1.5 µg of genomic DNA was randomly fragmented with a Covaris instrument. Fragments with sizes between 200 and 400 bp were subsequently selected with an Agencourt AMPure XP-Medium Kit, followed by end repair, 3′ adenylation, and adapter ligation. After PCR enrichment, the PCR products were recovered with the AxyPrep Mag PCR Clean-up Kit. The double-stranded PCR products were heat-denatured and circularized with the splint oligo sequence. Single-stranded circular DNA (ssCir DNA) was formatted as the final library and qualified according to quality control (QC) procedures. The qualified libraries were sequenced on the MGISEQ2000 platform at CNGB (Shenzhen, China).

RNA was extracted with a Qiagen RNA isolation kit, and the RNA quality was assessed by 0.75% agarose gel electrophoresis and an Agilent 2100 Bioanalyzer. The sequencing libraries were prepared with the MGIEasy RNA Directional Library Prep Kit (BGI, China) according to the manufacturer’s protocol, and 150 bp paired-end (PE) sequencing was performed with the MGISEQ2000 platform at CNGB (Shenzhen, China).

### 2.3. Transcriptome Assembly and Functional Annotation

We first filtered the raw sequencing reads before performing transcriptome assembly. The data were processed in the following manner: (1) the reads were removed if they contained adapters; (2) the reads were removed if the N ratio was greater than 10%, where N denotes base information that is undetermined; and (3) the low-quality reads, defined as those with a Qphred score of less than 20, were removed, which accounted for more than 50% of the total read length. The aforementioned processes were conducted for the quality control of the WGS and RNA sequencing data with fastp software (v0.21.0) [38]. De novo assembly of the transcriptome was performed with Trinity (v2.14.0) [39]. Following quality control procedures, each sample was assembled with the software SOAPdenovo2 (V2.04) [40]. Owing to technical limitations, many alternative splicing forms of the sequences assembled in the Trinity de novo transcript assembly could not be correctly merged, resulting in a relatively large number of genes. The gaps were subsequently filled with GapCloser (V1.10) [41].

The initial step was to utilize TransDecoder (V5.5.0) (https://github.com/TransDecoder/TransDecoder, accessed on 1 May 2024) to predict the sequence structure of the transcripts assembled by Trinity. The genomeThreader (V1.7.3) [42] was subsequently used to predict the gene structure of the sequence, which was then used to predict the gene structure. The transcript identified was the representative transcript of the sequence. On the basis of the correspondence between the Trinity genes and transcripts, the longest transcript was subsequently selected as the structural annotation result and the sequence of the locus. The protein sequences resulting from the final structural annotation were submitted for annotation to various databases, including the Kyoto Encyclopedia of Genes and Genomes (KEGG) database (http://www.genome.jp/kegg, accessed on 16 May 2024), the SwissProt database, and the NCBI nonredundant (NR) database. The domain, motif, and Gene Ontology (GO) annotations from these and other databases were determined with Iprscan (version 5.22) [43]. The BLASTp (http://www.ncbi.nlm.nih.gov/BLAST/, accessed on 29 June 2024) results from the NR and SwissProt databases were then subjected to further screening to identify the best alignment results. This was performed by applying the following conditions: identity ≥ 30, query coverage ≥ 30, and subject coverage ≥ 30.

### 2.4. Correlation Analysis

The correlation coefficient between samples was calculated with the script PtR, which is part of the Trinity software (version 2.14.0). A correlation coefficient approaching 1 indicated a stronger correlation between samples. Principal component analysis (PCA) used the PtR script of Trinity (v2.14.0) to generate the PCA graph. Concurrently, a t-distributed stochastic neighbor embedding (t-SNE) representation was generated and rendered on the basis of the Rtsne and ggplot2 packages in R (v4.1.3) (https://www.R-project.org/).

### 2.5. Differential Expression Analysis

The Trinity software package (version 2.14.0) was used to run the script, with the parameter set to ‘edgeR’ to perform the necessary gene expression difference analysis [44]. The adjusted *p*-values were used for the false discovery rate (FDR), and the genes were subjected to manual filtering. The threshold for this was set at |LogFC| ≥ 1 and *p* < 0.05/|LogFC| ≥ 2 and *p* < 0.001. A greater difference between the genes in the samples was indicated by a larger absolute value of logFC and a smaller *p*-value. The selected differentially expressed genes were subjected to further analysis.

### 2.6. Single Amino Acid Polymorphism (SAAP) Analysis of Scale Color Differences

The genes associated with body color were sourced from the SwissProt database, and candidate genes were identified through a BLASTp comparison. The database assembled in the second part was derived from the literature [45], and candidate genes were identified through a TBLASTn comparison. OrthFinder2 software (v2.4.0) [46] was used to identify orthologs of genes present within the genomes of the two species, specifically *L. rufozonatus* and *L. rosozonatus*. OMA (Orthologous Matrix) (v2.4.1) [47] was used to identify homologous gene matrices and conduct a phylogenetic analysis. The genomic data of the two species were subjected to processing, resulting in the construction of a gene homology matrix.

## 3. Results

### 3.1. Whole-Genome Transcriptome Sequencing and Assembly

**Figure 2:**
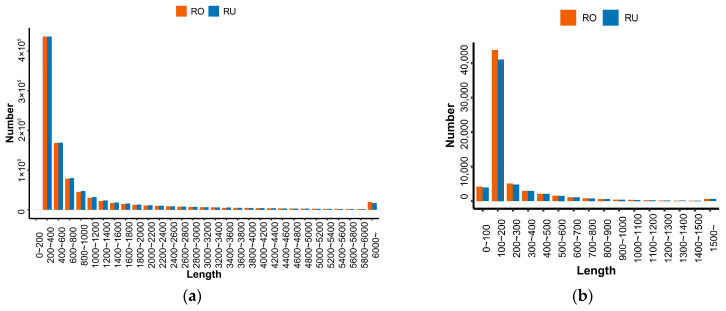
Information about the transcripts obtained by sequencing. (**a**) Distribution of transcript length. (**b**) Distribution of the longest transcript protein length. RO: *L. rosozonatus*; RU: *L. rufozonatus*.

Totals of 813,471,692 and 1,100,930,340 DNA raw reads were generated from the MGISEQ2000 platform for *L. rufozonatus* and *L. rosozonatus*, respectively. Following quality control filtering, 721,023,740 and 975,554,490 DNA clean reads were obtained for these two species, resulting in 88.64% and 88.61% high-quality reads used for whole-genome assembly (Table S1). Similarly, 361,462,332 and 341,960,483 RNA raw reads were generated from the MGISEQ2000 platform for *L. rufozonatus* and *L. rosozonatus*, respectively. After quality control filtering, 350,346,591 and 331,537,523 RNA clean reads were acquired for these two species, with 96.92% and 96.95% high-quality reads utilized for transcriptome assembly (Table S2). After the Trinity assembly of the cleaned RNA-Seq data, 957,571 transcripts and 581,534 unigenes were obtained from *L. rufozonatus*, whereas 949,552 transcripts and 603,244 unigenes were obtained from *L. rosozonatus* (Figure 2a).

### 3.2. Annotation Analysis

For predicting the assembled genes, the longest transcript was chosen as the representative sequence for the locus. The protein length of the longest transcript in both species ranged primarily from 100 to 200 bp (Figure 2b). The proportion of completely aligned genes was 91.68% for *L. rufozonatus* and 92.01% for *L. rosozonatus*, indicating that the prediction results were relatively comprehensive (Table 1).

The protein sequences of the final structural annotation results were annotated to obtain 60,852 gene datasets from *L. rufozonatus* and 64,209 gene datasets from *L. rosozonatus*. The results indicated that there were 25,522 (93.5%), 16,516 (60.5%), 12,723 (46.6%), 20,961 (76.8%), and 15,018 (55%) genes in *L. rufozonatus* that matched the Iprscan, Gene Ontology (GO), Kyoto Encyclopedia of Genes and Genomes (KEGG), nonredundant (NR), and SwissProt databases. In contrast, *L. rosozonatus* presented 28,860 (94.1%), 18,561 (60.5%), 13,499 (44%), 22,319 (72.8%), and 15,737 (51.3%) genes matching the same databases. The total numbers of successfully annotated genes were 27,281 (44.8%) for *L. rufozonatus* and 30,637 (47.7%) for *L. rosozonatus* (Table 2).

**Figure 3:**
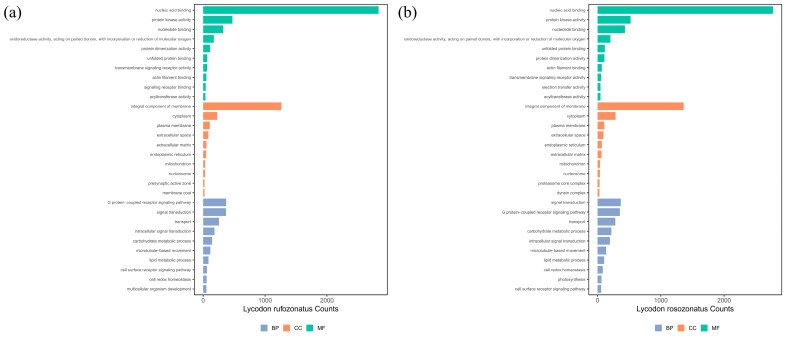
(**a**) Histogram of Gene Ontology (GO) classification statistics of *L. rufozonatus*. (**b**) Histogram of Gene Ontology (GO) classification statistics of *L. rosozonatus*. The horizontal axis shows the number of genes in different GO entries (functional categories); the vertical axis shows the specific category name of GO functional classification. Green is for molecular function (MF), orange is for cell composition (CC), and blue is for biological process (BP).

**Figure 4:**
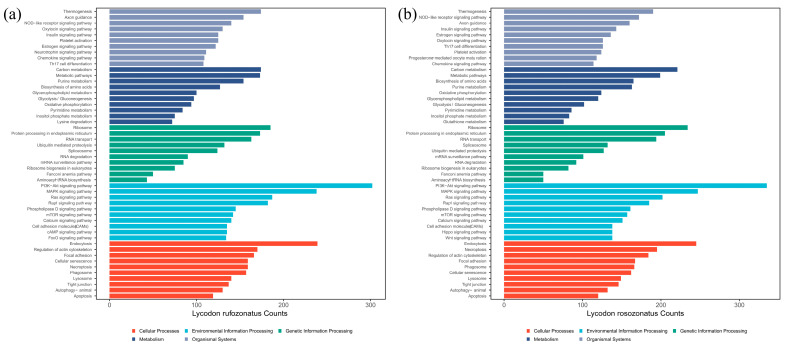
(**a**) Bar chart of KEGG pathway classification statistics of *L. rufozonatus*. (**b**) Bar chart of KEGG pathway classification statistics of *L. rosozonatus*. The horizontal axis shows the number of genes involved in the pathway, and the vertical axis shows the name of the specific KEGG pathway. The colors are as follows: red (cellular processes); green (genetic information processing); blue–green (environmental information processing); dark blue (metabolism); light blue (organismal systems).

The genes successfully assigned to GO terms were classified into three categories: cellular components (CCs) (24.8% for RO and 23.4% for RU), molecular functions (MFs) (25.8% for RO and 24.7% for RU), and biological processes (BPs) (49.4% for RO and 51.9% for RU) based on their putative functions (Figure 3). Among these, the largest proportion of genes in the biological process category was related to metabolic processes and cellular processes, whereas the cellular component category was dominated by genes associated with membrane and organelle components. The molecular function category primarily included genes related to catalytic and binding activities. KEGG pathway classification assigned 13,499 genes for *L. rosozonatus* and 12,723 genes for *L. rufozonatus* to various pathways. These pathways were grouped into five major categories: metabolism, genetic information processing, environmental information processing, cellular processes, and organismal systems. Among them, the most abundant term was signal transduction in the environmental information processing category, followed by pathways related to the endocrine system, immune system, cellular community, and transport and catabolism (Figure 4). These results highlight the diverse functional roles of genes in regulating biological processes and maintaining organismal homeostasis.

### 3.3. Correlation Analysis of Scales of Different Colors

**Figure 5:**
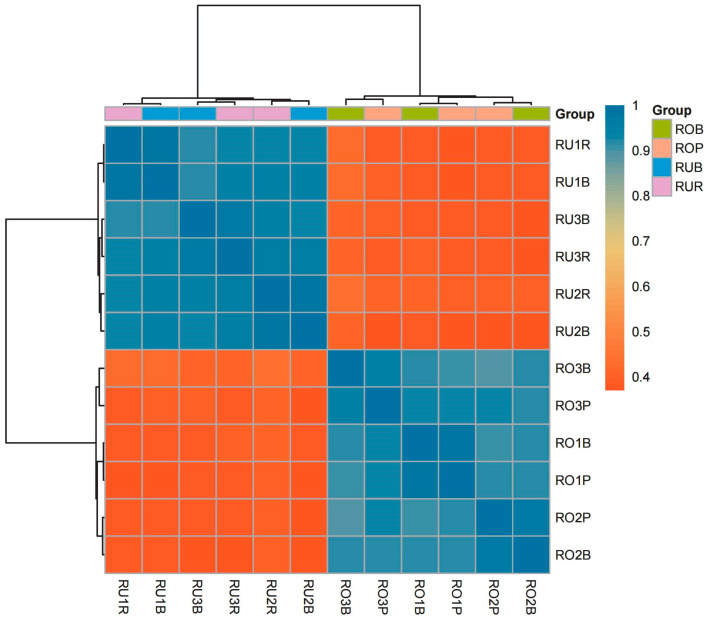
Correlation heatmap. The correlation of biological replicates was close to 1, indicating high consistency between samples (blue indicates high correlation; red indicates low correlation). ROB: black skin scales of *L. rosozonatus*; ROP: pink skin scales of *L. rosozonatus*; RUB: black skin scales of *L. rufozonatus*; RUR: red skin scales of *L. rufozonatus*; 1, 2, and 3 represent different individual numbers.

The colored scales from different regions of the three *L. rufozonatus* were categorized into six groups (RU1R, RU1B, RU2R, RU2B, RU3R, and RU3B). Similarly, the colored scales from distinct regions of the three *L. rosozonatus* were categorized into six groups (RO1P, RO1B, RO2P, RO2B, RO3P, and RO3B). As illustrated in the sample correlation heatmap, the samples naturally clustered by species (*L. rufozonatus* and *L. rosozonatus*) in a manner that was readily apparent. Furthermore, the cluster tree at the top of the heatmap provided a clear delineation within the RU and RO samples, which were distributed across two main branches (Figure 5). A discernible grouping trend was evident in the gene expression patterns of the *L. rufozonatus* (RU) and *L. rosozonatus* (RO) samples. The correlation between the *L. rufozonatus* (RU1, RU2, and RU3) and *L. rosozonatus* (RO1, RO2, and RO3) samples was high, as indicated by the dark blocks within the group. In contrast, the correlation between *L. rufozonatus* (RU) and *L. rosozonatus* (RO) was low.

**Figure 6:**
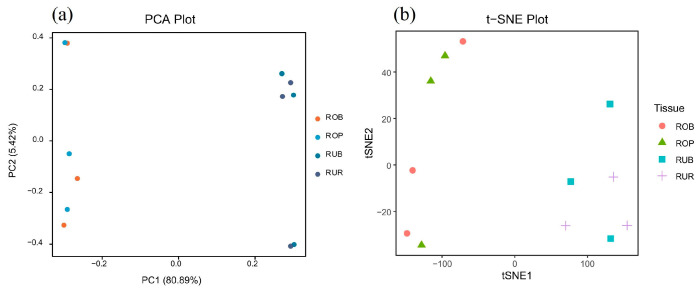
Dimensionality reduction analysis. (**a**) Principal component analysis (PCA): samples from different groups were grouped together well on the PC1 and PC2 axes, showing that the main differences between the samples were seen in the experiment. (**b**) t-distributed stochastic neighbor embedding (t-SNE): t-SNE dimensionality reduction analysis further confirmed the grouping of the samples, showing significant differences between species and the similarity of the data within groups. ROB: black skin scales of *L. rosozonatus*; ROP: pink skin scales of *L. rosozonatus*; RUB: black skin scales of *L. rufozonatus*; RUR: red skin scales of *L. rufozonatus*.

The samples were divided into four groups as follows: red scales of *L. rufozonatus* (RUR), black scales of *L. rufozonatus* (RUB), pink scales of *L. rosozonatus* (ROP), and black scales of *L. rosozonatus* (ROB). The results of the t-SNE analysis and PCA showed that scales of different colors within the same snake species were clustered (RUB and RUR clusters; ROB and ROP clusters), whereas scales of different colors from different species exhibited clear separation (the RU series of *L. rufozonatus* and the RO series of *L. rosozonatus* were distinctly separated) (Figure 6).

### 3.4. DEGs in Different Transcriptomes

To compare the differential expression patterns between RUR and RUB, ROP and ROB, and RUR and ROP, the analysis of the differentially expressed genes (DEGs) was performed using the Trinity (v2.14.0) package script run_DE_analysis.pl with the method edgeR parameter. Genes were manually filtered based on the following thresholds: |LogFC| ≥ 1 with *p* < 0.05, or |LogFC| ≥ 2 with *p* < 0.001. First, we compared the differentially expressed genes (DEGs) between the red and black scales of *L. rufozonatus* (RUR vs. RUB), as well as between the pink and black scales of *L. rosozonatus* (ROP vs. ROB). The differential expression analysis revealed that two genes were significantly upregulated and one gene was downregulated in RUB compared with RUR (Figure S1). In the case of ROB, 2 genes were found to be significantly upregulated and 192 genes were identified as downregulated in comparison with ROP (Figure S2). We conducted cluster analysis on scales from both snake species and found that the RU (RUB, RUR) and RO (ROB, ROP) samples were primarily grouped into two distinct clusters, indicating interspecies differences in gene expression (Figure S3).

**Figure 7:**
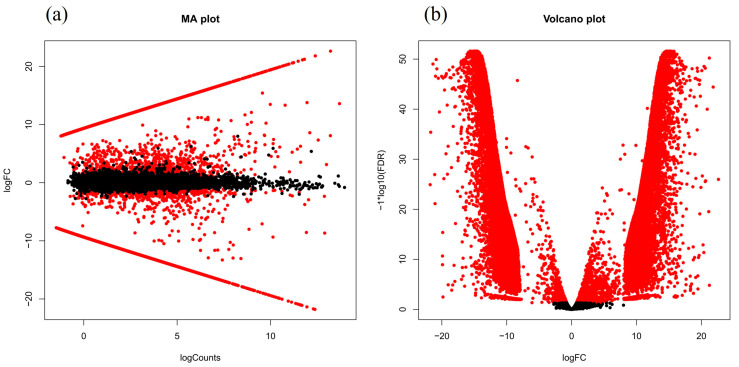
Analysis of genes with different expressions between RUR and ROP. (**a**) MA plot: The horizontal axis (logCounts) shows the average expressions of genes, and the vertical axis (logFC) shows the logarithmic change in the gene expression. Positive logFC values indicate the upregulation of ROP genes relative to RUR genes, and negative values indicate the downregulation of genes (*p* < 0.05). (**b**) Volcano plot: The horizontal axis (logFC) represents the logarithmic fold change of genes, the left side of the horizontal axis shows genes downregulated by ROP relative to RUR, and the right side shows upregulated genes. The vertical axis (−log10(FDR)) represents the significance of differentially expressed genes; the higher the value, the more significant the differential expression of the genes (*p* < 0.05) Red dots represent significantly differentially expressed genes, while black dots represent non-significantly differentially expressed genes.

**Figure 8:**
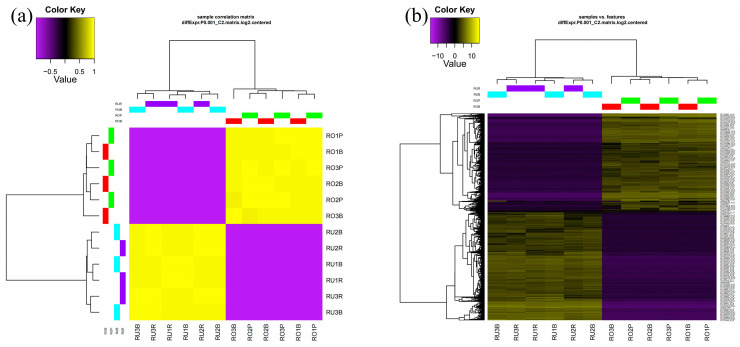
Correlation heatmap and expression heatmap of differentially expressed genes. (**a**) Correlation heatmap: The horizontal and vertical axes represent different samples (e.g., RO1P, RU2R, etc.). The color key ranges from purple (negative correlation, −0.5) to yellow (positive correlation, +1). The yellow area shows a high positive correlation between samples, while purple shows a negative or low correlation. The color of the cells in the heatmap shows the correlation between two samples (Pearson correlation coefficient). (**b**) Expression heatmap: the horizontal axis represents the samples, and the vertical axis represents the names of the genes that were found to be expressed, from purple (low expression, −10) to yellow (high expression, +10).

Finally, we conducted a comparison of the DEGs (RUR vs. ROP) between the pink and red scales of *L. rufozonatus* and *L. rosozonatus*. The results of the differential analysis demonstrated that 12375 genes were significantly upregulated and 13599 gene were significantly downregulated in ROP relative to RUR (Figure 7). A cluster analysis of ROP and RUR revealed that there were 25,974 genes whose expressions significantly differed between the two species, which may reflect the large differences in the scale coloration and pigmentation-related pathways between *L. rufozonatus* and *L. rosozonatus*. Such differences in the production of different scale colors in the two species (Figure 8) further emphasize their distinct gene expression profiles, which may be driven by adaptive evolution in response to different ecological or physiological pressures.

### 3.5. SAAP Analysis of Differentially Expressed Genes

A total of 1,709 genes associated with body color were identified through a BLAST comparison. The differentially expressed genes (DEGs) were analyzed with a candidate set of body color-related genes, and the significantly differentially expressed genes related to body color were identified. The expressions of four genes significantly differed between ROB and ROP (Table 3). Three of the genes (Unigene9453 (*shroom2*), Unigene11641 (*shroom2*), and Unigene9433 (*shroom2*)) are members of the Shroom protein family, which is predominantly involved in regulating cell morphology. The Unigene9417 (*notch1*) gene represents a crucial element within the Notch signaling cascade. This pathway plays a pivotal role in regulating cellular differentiation, proliferation, and apoptosis. Moreover, *notch1* is a key mediator in the development and maintenance of melanocytes, influencing pigment synthesis and deposition. In addition, in the indirect interaction of signaling pathways, *shroom2* regulates cell contraction by binding to Rho-associated coiled-coil containing protein kinase (ROCK), while *notch1* activation can regulate the activity of Rho GTPases, which may affect the function of the *shroom2*-ROCK complex. In the transcriptional regulatory network, *notch1* target genes (such as the HES/HEY family) may regulate the expression of *shroom2*.

**Figure 9:**
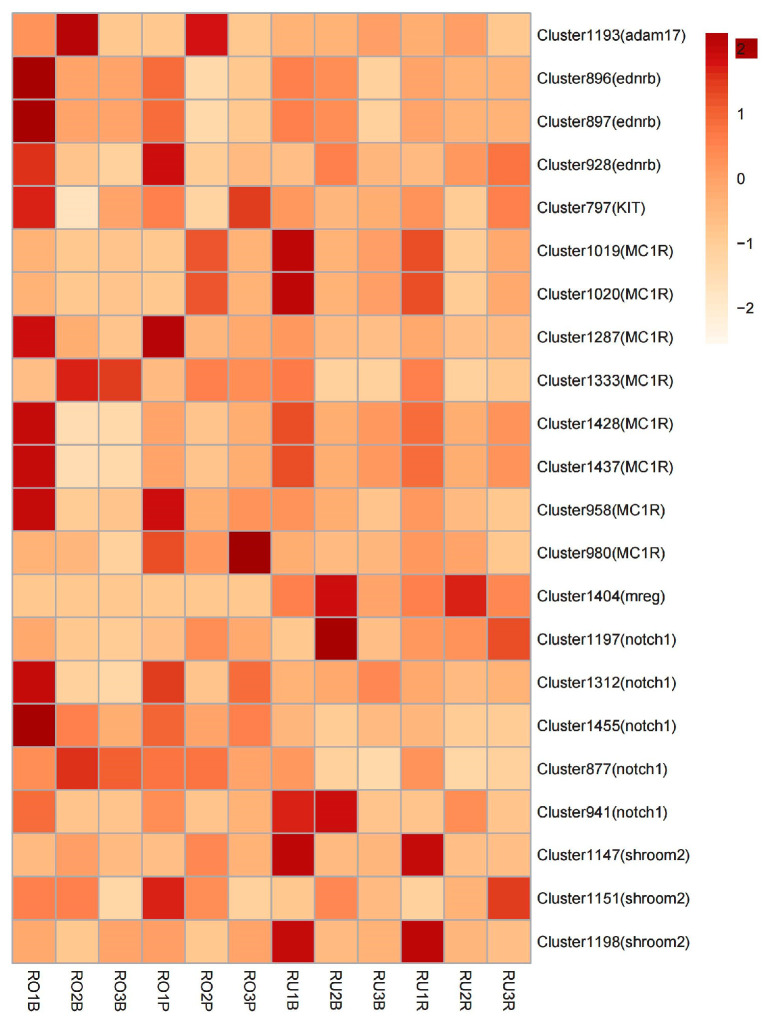
Gene expression heatmap. The horizontal axis shows sample numbers, and the vertical axis shows gene clusters. The color legend shows the range of values from light yellow (low expression or negative values) to dark red (high expression or positive values).

Among these differentially expressed genes, a comprehensive analysis of the single-copy homologous genes and amino acid protein sequences of five species revealed the identification of common body color-related genes shared by *L. rufozonatus* and *L. rosozonatus*. This analysis also identified 22 common gene loci that had mutated (Table S3). These included eight loci in the *MC1R* gene, eight loci in the *shroom2* gene, five loci in the *notch1* gene, five loci in the *ednrb* gene, one locus in the *adam17* gene, one locus in the *mreg* gene, and one locus in the *KIT* gene (Table 4). Concomitantly, the expression levels of the genes located at these mutation sites exhibited varying degrees of difference between the different scale color groups of the two species (Figure 9). The consensus color-associated genes and differentially expressed genes were identified through their overlap to identify genes exhibiting significantly altered expression and SAAP. Consequently, three genes with notable discrepancies, RU_DN1145_c3_g2 (*mreg*), RU_DN10511_c0_g1 (*notch1*), and Unigene11172 (*notch1*), were identified as genes of particular importance (Table 5). Melanoregulin (*mreg*) is a protein that is involved in the transport and release of melanosomes. It regulates the melanin transport pathway, particularly in melanocytes, and is responsible for the distribution and export of melanin granules. The expression of the *mreg* gene was markedly downregulated in ROP (logFC = −15.19, FDR = 1.39 × 10^−35^), suggesting its potential involvement in the regulation of diverse body color phenotypes. The expression of the RU_DN10511_c0_g1 (*notch1*) gene was significantly downregulated in the ROP samples (logFC = −10.83, FDR = 7.40 × 10^−27^), which may result in a reduction in the melanocyte differentiation capacity.

## 4. Discussion

The study of animal color patterns is a long-standing and significant area of research within the field of evolutionary biology, with squamates representing a particularly diverse group in this regard [48]. Some venomous snakes use their bright colors as a form of warning, whereas nonvenomous snakes that are more distantly related to them imitate the colors of these venomous snakes as a means of avoiding the risk of predation [49], such as the venomous coral snake (*Micrurus alleni*) and the nonvenomous Colubrid mimic (*Oxyrhopus petolarius*). As a consequence of this evolutionary process, these animals are better equipped to survive in their environments. This finding demonstrates that animals can utilize their distinctive scale color to achieve two key objectives: first, blending into their surroundings, thereby avoiding detection by predators, and second, creating a visual distraction that impairs the predator’s ability to track and identify their prey [50,51]. This study examined the differences in the scale coloration between two closely related snake species, providing valuable insights into the pigmentation mechanisms in non-model animals. Concurrently, investigating the genetic basis of animal color disparities offered significant insights into the study of phenotypic diversity, species divergence, and adaptive evolution.

The coloration observed in reptiles is the result of two distinct processes: the reflection and scattering of light by cells and tissues (structural coloration) and the absorption of light by chemical pigments within dermal chromatophore cells, of which there are three main types [52]. The uppermost layer of chromatophores is composed of xanthophores, which contain yellow–red pigments. The iridophores, which lie beneath the xanthophores, contain colorless crystals of guanine. These reflect and scatter light, and the resulting color is dependent on the size and spacing of the platelets. The deepest layer is comprised of melanophores, which contain black eumelanin pigments (pheomelanins have not been identified in reptiles). These absorb all the remaining wavelengths of light [33]. The color of a given patch of skin in lizards and snakes is determined not only by the static structural combination of pigment cells [53] but also by dynamic interactions between chemical and physical parameters [8,54]. For example, Teyssier et al. [54] demonstrated that chameleons are capable of rapidly altering their color through the active tuning of a lattice of guanine nanocrystals within a superficial, thick layer of dermal iridophores. Collectively, the collective interactions of pigment cells give rise to not only a diverse spectrum of visible colors but also a range of physiological functions.

In the present study, a comparative transcriptomic analysis of the red scales and pink scales of *L. rufozonatus* and *L. rosozonatus* was conducted, resulting in the identification of 25,974 significantly differentially expressed genes (DEGs). A comprehensive analysis of the single amino acid polymorphism (SAAP) dataset was performed. This analysis revealed the existence of mutation sites within a number of pigmentation genes (*MC1R*, *shroom2*, *notch1*, *ednrb*, *adam17*, *mreg*, and *KIT*) that were differentially expressed between the two samples. The *MC1R* gene is responsible for the dysfunction of melanin synthesis, which can be observed in lizards inhabiting white sand dunes and displaying whitish coloration [27]. Similar findings have been reported in *Oophaga histrionica*, a species of poison frog. In this species, mutations in the *MC1R* gene, which affects melanogenesis, have resulted in the production of a lighter, more brownish background in some populations [55]. Notably, Saenko et al. [56] postulated that despite the inhibition of melanin synthesis, melanophores devoid of melanin can nevertheless perform pivotal functions in the process of pattern formation. The development of body color is the consequence of the aggregation and manifestation of a multitude of body color genes. Among the genes related to melanin, *KIT* plays a crucial role in the melanogenesis signaling pathway. The mutation or deletion of *KIT* can result in the development of different hair and skin colors in mammals [57]. The melanogenesis process can be stimulated by stem cell factor/c-kit signaling in normal human epidermal melanocytes when they are exposed to norepinephrine [58]. *KIT* regulates a number of processes in melanocytes, including cell migration, survival, proliferation, and differentiation [59]. Furthermore, it interacts synergistically with *MC1R* [60]. These two genes have different mutation sites, which may have resulted in disparate expression levels in the melanocytes in the scales of the two species.

For example, a notable proportion of color genes with locus mutations in our dataset are integral components of the tyrosinase pathway (*MC1R*, *adam17*, and *ednrb*). This pathway is subject to enzymatic regulation by tyrosinase, in addition to other enzymes and cofactors, and is instrumental in the synthesis of melanin [61]. *ednrb* has been shown to be involved in the production of the different male color morphs of the ruff (a sandpiper) and is expressed only in black males [62]. This same phenomenon was identified in a study of the skin color of four poison frogs: *ednrb* was not expressed in the blue–black morph, and only one of the *ednrb* transcripts was expressed in the San Felix morph [45]. Mutations in *ednrb* affect pigment cell development (especially in melanocytes and iridophores) in a variety of vertebrate species [63]. In the two snakes studied, eight sites in *shroom2* were found to be mutated, indicating that this gene had the greatest number of mutation sites. In some studies, *shroom2* was found to regulate the generation and localization of melanosomes in retinal pigment epithelial cells. The absence of the *shroom2* function has been demonstrated to result in the obstruction of melanosome maturation and affect its localization at the top of the cell. Conversely, the overexpression of *shroom2* has been shown to induce the accumulation of melanin granules on the apical surfaces of epithelial cells [64]. Furthermore, they have been demonstrated to play a role in the pigmentation of oocytes in amphibians. The changes in the localization and expression levels of *shroom2* and Spectralin are closely related to the diversity of oocyte pigment patterns [65]. Mutations in these loci, which are responsible for the expression of the skin color gene, clearly result in alterations in the color of the animal.

*Notch* signaling is a highly conserved signaling pathway that is involved in various biological processes, including organ formation and tissue repair and function [66]. This study examined the differential expression levels of the *notch1* gene, a prominent member of the *Notch* signaling pathway. The results revealed that the expression levels of the *notch1* gene varied between red and pink scales. Mutations in this gene can influence human skin, hair, and eye pigmentation through effects on melanocyte stem cells [67]. *Notch1* was also found to be differentially expressed between color morphs in the study by Stuckert et al., and the differential expression in combination with *srm* and *rtf1* SNPs suggests that these genes may play a role in the divergence of pattern elements between color morphs [45]. At the same time, melanoregulin (*mreg*), a product of the dilute suppressor gene, has been implicated in the regulation of melanosome transport in mammalian epidermal melanocytes [68,69]. This is supported by the finding that *mreg* deficiency restores peripheral melanosome distribution from perinuclear melanosome aggregation in Rab27A-deficient melanocytes [70,71,72]. Similarly, melanocytes from mice that are deficient in myosin Va and thus exhibit reduced levels of myosin Va [73] demonstrate a marked redistribution of melanosomes from the dendritic tips to the cell center. This redistribution leads to a reduction in intercellular melanosome transfer, resulting in a reduction in or ‘dilution’ of the mouse’s coat color [74]. For example, mice that are genetically black but also homozygous for a functional null allele at the dilute locus appear gray [75]. Our results revealed that the RU_DN1145_c3_g2 (*mreg*) and RU_DN10511_c0_g1 (*notch1*) genes were significantly downregulated in pink scales compared with red scales. The substantial disparity in coloration exhibited by these two snakes was attributable to the differential expression of two genes that are associated with color regulation. To this end, we used transcriptomics to provide a molecular basis for the difference in the scales between these two snakes. This provides a foundation for the subsequent integration of multiomics and multiangle explanations of this phenomenon.

## 5. Conclusions

In summary, a high-quality transcriptome dataset was assembled with the objective of providing insight into the genetic basis of the scale color differences between *L. rufozonatus* and *L. rosozonatus*. Our results revealed that the mutation sites of the common body color genes in the two species exhibited variations at disparate sites within seven genes. Furthermore, the data were subjected to dimensionality reduction analysis, which revealed a significant separation between the scales of the two snakes. Concurrently, the black scales of *L. rufozonatus* were closely associated with the red scales, whereas the black scales of *L. rosozonatus* were closely associated with the pink scales. A comparative analysis of the common color-related genes and the differentially expressed genes revealed that two genes, *mreg* and *notch1*, were notably downregulated in *L. rosozonatus* compared with *L. rufozonatus*. Therefore, we speculate that the downregulation of these two genes affects melanosome differentiation and release, leading to changes in the scale color in these two species. However, the ecological fitness of the observed variations in the skin color remains unclear. Further observations and analyses are needed to ascertain the adaptive significance of the color variation.

## Figures and Tables

**Table 1:** Integrity assessment statistics.

Group ^1^	RO Trinity	RU Trinity	RO Protein Coding	RU Protein Coding
C	92.01%	91.68%	91.68%	91.32%
S	24.78%	23.52%	24.75%	23.43%
D	67.23%	68.16%	66.94%	67.89%
F	4.03%	4.38%	3.79%	4.20%
I	0.09%	0.00%	0.09%	0.03%
M	3.88%	3.94%	4.44%	4.44%

^1^ C (Complete Genes): the Benchmarking Universal Single-Copy Orthologs (BUSCO) genes that can be completely aligned in the assembly; S (Single-Copy Complete Genes): the BUSCO genes that can be entirely aligned in the assembly, with only one copy present; D (Duplicated Complete Genes): the BUSCO genes that can be completely aligned in the assembly, with more than one copy present; F (Fragmented Genes, subclass 1): the BUSCO genes in which only a portion of the gene is present in the assembly, and the rest of the gene cannot be aligned; I (Fragmented Genes, subclass 2): the BUSCO genes in which a section of the gene aligns to one position in the assembly, while the remaining part aligns to another position; M (Missing Genes): the BUSCO genes with no alignment present in the assembly.

**Table 2:** Functional annotation statistics.

Database	RO	RU
Iprscan	28,860	25,522
GO	18,561	16,516
KEGG	13,499	12,723
NR	22,319	20,961
SwissProt	15,737	15,018
Annotated	30,637	27,281
Unannotated	33,572	33,571
Total	64,209	60,852

**Table 3:** Table of genes related to body color.

GeneID	Group	LogFC	*p*-Value	FDR
Unigene9453 (*shroom2*)	ROB_vs_ROP	−5.410569297	1.02 × 10^−6^	0
Unigene11641 (*shroom2*)	ROB_vs_ROP	−3.182307072	5.68 × 10^−6^	0
Unigene9433 (*shroom2*)	ROB_vs_ROP	−4.081419141	3.39 × 10^−5^	0
Unigene9417 (*notch1*)	ROB_vs_ROP	−4.479233533	8.00 × 10^−5^	0.01

**Table 4:** Shared gene annotation and variant site information.

GroupID	GeneName	Counts	Mutation
Cluster1019	*MC1R*	1	V144A
Cluster1020	*MC1R*	1	S135N
Cluster1147	*shroom2*	2	T389P, N1086T
Cluster1151	*shroom2*	5	E586D, N637S, E660K, T756A, T776M
Cluster1193	*adam17*	1	D26E
Cluster1197	*notch1*	1	H90R
Cluster1198	*shroom2*	1	S530P
Cluster1287	*MC1R*	1	V133I
Cluster1312	*notch1*	1	H27Q
Cluster1333	*MC1R*	1	A101V
Cluster1404	*mreg*	1	V14A
Cluster1428	*MC1R*	1	L42F
Cluster1437	*MC1R*	1	S69P
Cluster1455	*notch1*	1	N749S
Cluster797	*KIT*	1	P85A
Cluster877	*notch1*	1	E21A
Cluster896	*ednrb*	2	Y42F, D68N
Cluster897	*ednrb*	1	K179R
Cluster928	*ednrb*	2	L202F, L307V
Cluster941	*notch1*	1	Y25H
Cluster958	*MC1R*	1	H46Q
Cluster980	*MC1R*	1	L226S

**Table 5:** The common body color-related genes and differentially expressed genes of *L. rufozonatus* and *L. rosozonatus* were intersected to obtain an expression table of the intersection genes.

GeneID	Sample A	Sample B	logFC	Log Counts per Million (logCPM)	Probability Value (*p*-Value)	FDR
RU_DN1145_c3_g2 (*mreg*)	ROP	RUR	−15.19373837	5.77320102074195	1.3015005339553 × 10^−36^	1.38737854091305 × 10^−35^
RU_DN10511_c0_g1 (*notch1*)	ROP	RUR	−10.82609391	1.42891518860247	1.30325616007406 × 10^−27^	7.40272367248823 × 10^−27^
Unigene11172 (*notch1*)	ROP	RUR	2.26713514896129	1.73494640766586	5.02842825852524 × 10^−5^	7.21132394782175 × 10^−5^

## Data Availability

Transcriptome data are available in the NCBI, and the link to the data is https://www.ncbi.nlm.nih.gov/bioproject/PRJNA1224317, accessed on 18 March 2025.

## Supplementary Materials

The following supporting information can be downloaded at https://www.mdpi.com/article/10.3390/ani15071061/s1, Figure S1. Differences in gene expression between red and black scales of *L. rufozonatus*. (a) MA plot: The horizontal axis (logCounts) shows the average expression of genes, and the vertical axis (logFC) shows the logarithmic change in gene expression, where a positive logFC value indicates that the gene was upregulated in the experimental group, and a negative value indicates that the gene was downregulated (*p* < 0.05). (b) Volcano plot: The horizontal axis (logFC) represents the logarithmic fold change of genes, with downregulated genes on the left side of the horizontal axis and upregulated genes on the right side. The vertical axis (−log10(FDR)) represents the significance of differentially expressed genes, and the higher the value, the more significant the differential expression of genes (*p* < 0.05). Figure S2. Differences in gene expression between pink and black scales of *L. rosozonatus*. (a) MA plot: The horizontal axis (logCounts) shows the average expression of genes, and the vertical axis (logFC) shows the logarithmic change in gene expression, where a positive logFC value indicates that the gene was upregulated in the experimental group, and a negative value indicates that the gene was downregulated (*p* < 0.05). (b) Volcano plot: The horizontal axis (logFC) represents the logarithmic fold change of genes, with downregulated genes on the left side of the horizontal axis and upregulated genes on the right side. The vertical axis (−log10(FDR)) represents the significance of differentially expressed genes, and the higher the value, the more significant the differential expression of genes (*p* < 0.05). Figure S3. Correlation heat map between the two species. The horizontal and vertical axes represent different samples (e.g., RO1P, RU2R, etc.). The color key ranges from purple (negative correlation, −0.5) to yellow (positive correlation, +1). The yellow area shows a high positive correlation between samples, while purple shows negative or low correlation. The color of the cells in the heatmap shows the correlation between two samples (Pearson correlation coefficient). Table S1: Sample whole genome assembly results. Table S2: Sample skin scale transcriptome assembly results. Table S3: Mutation information of common gene mutation sites.
