# Supplementary material for: Comparison of Transcriptome Differences in Scales of Two Closely Related Snake Species (Lycodon rufozonatus and Lycodon rosozonatus)
Source: Animals (Basel). 2025 Apr 6;15(7):1061. doi: 10.3390/ani15071061 (PMC11988092; doi:10.3390/ani15071061)
Supplement: Supplementary file 1 [file animals-15-01061-s001.zip › Figures S1, S2, S3.pdf]

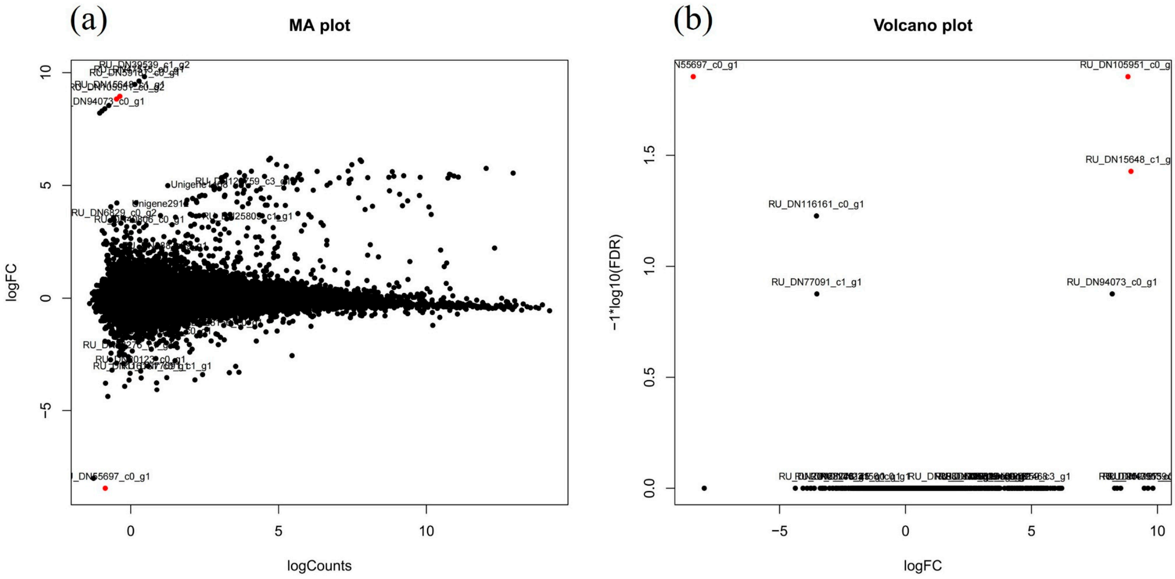

Figure S1. Differences in gene expression between red and black scales of *L. rufozonatus*. (a) MA plot: The horizontal axis (logCounts) shows the average expression of genes, and the vertical axis (logFC) shows the logarithmic change in gene expression, where a positive logFC value indicates that the gene was upregulated in the experimental group, and a negative value indicates that the gene was downregulated ($P < 0.05$). (b) Volcano plot: The horizontal axis (logFC) represents the logarithmic fold change of genes, with downregulated genes on the left side of the horizontal axis and upregulated genes on the right side. The vertical axis (-log10(FDR)) represents the significance of differentially expressed genes, and the higher the value, the more significant the differential expression of genes ($P < 0.05$).

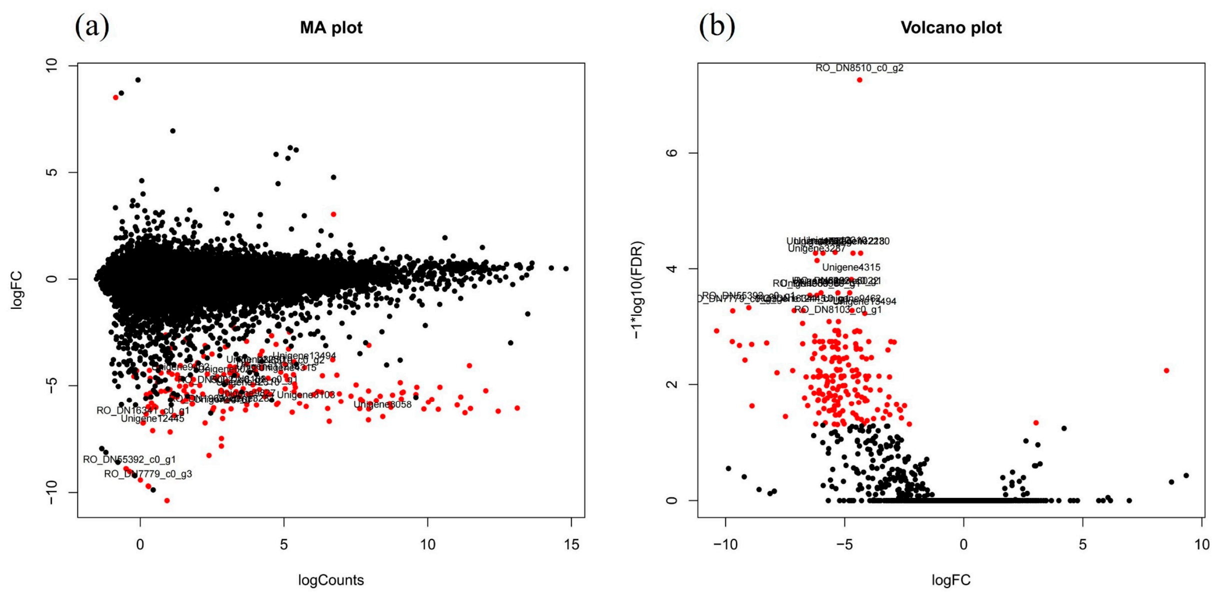

Figure S2. Differences in gene expression between pink and black scales of *L. rosozonatus*. (a) MA plot: The horizontal axis (logCounts) shows the average expression of genes, and the vertical axis (logFC) shows the logarithmic change in gene expression, where a positive logFC value indicates that the gene was upregulated in the experimental group, and a negative value indicates that the gene was downregulated ($P < 0.05$). (b) Volcano plot: The horizontal axis (logFC) represents the logarithmic fold change of genes, with downregulated genes on the left side of the horizontal axis and upregulated genes on the right side. The vertical axis (-log10(FDR)) represents the significance of differentially expressed genes, and the higher the value, the more significant the differential expression of genes ($P < 0.05$).

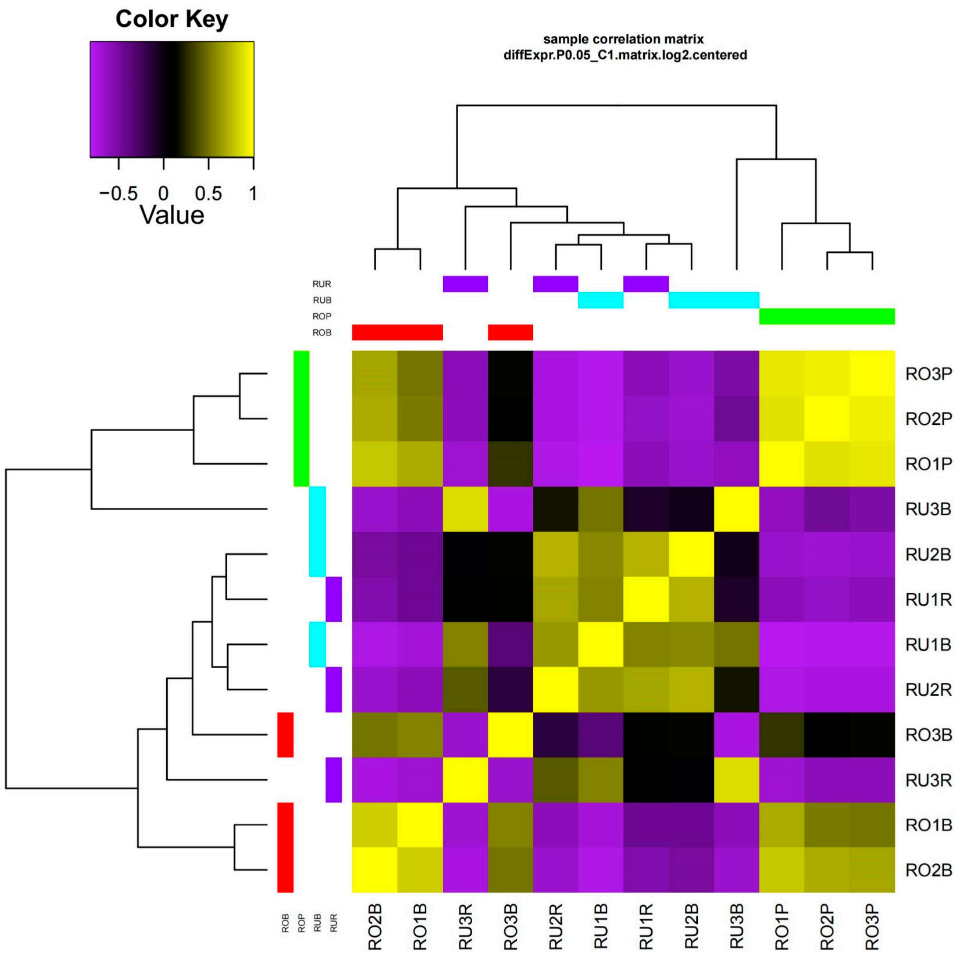

Figure S3. Correlation heat map between the two species. The horizontal and vertical axes represent different samples (e.g. RO1P, RU2R, etc.). The color key ranges from purple (negative correlation, -0.5) to yellow (positive correlation, +1). The yellow area shows a high positive correlation between samples, while purple shows negative or low correlation. The color of the cells in the heatmap shows the correlation between two samples (Pearson correlation coefficient).
