# Supplementary material for: Comparison of Transcriptome Differences in Scales of Two Closely Related Snake Species (Lycodon rufozonatus and Lycodon rosozonatus)
Source: Animals (Basel). 2025 Apr 6;15(7):1061. doi: 10.3390/ani15071061 (PMC11988092; doi:10.3390/ani15071061)
Supplement: Supplementary file 1 [file animals-15-01061-s001.zip › supplementary Tables S1, S2, S3.pdf]

Table S1: Sample whole genome assembly results

Samples	Raw Reads	Raw Bases(bp)	Clean Reads	Clean Bases(bp)	Rate(%)
RO1	272,032,135	81,609,640,500	235,373,406	64,221,327,170	86.52
RO2	297,698,430	89,309,529,000	269,228,705	73,928,307,133	90.44
RO3	243,741,127	73,122,338,100	216,421,629	59,336,141,954	88.79
RU1	275,752,676	82,725,802,800	240,014,345	65,568,437,662	87.04
RU2	307,666,295	92,299,888,500	270,860,987	74,248,096,046	88.04
RU3	263,107,213	78,932,163,900	232,980,566	63,785,861,449	88.55
RU4	254,404,156	76,321,246,800	231,698,592	63,702,800,188	91.08

Table S2: Sample skin scale transcriptome assembly results

Samples	Raw Reads	Raw Bases(bp)	Clean Reads	Clean Bases(bp)	Rate(%)
RO1P	62,542,273	18,762,681,900	60,439,880	16,611,486,490	96.64
RO1B	55,178,789	16,553,636,700	53,599,343	14,763,451,100	97.14
RO2P	63,102,781	18,930,834,300	61,197,324	16,813,549,985	96.98
RO2B	60,824,231	18,247,269,300	59,070,618	16,219,436,693	97.12
RO3P	59,116,092	17,734,827,600	57,226,887	15,695,916,921	96.8
RO3B	60,698,166	18,209,449,800	58,812,539	16,148,530,236	96.89
RU1B	58,449,719	17,534,915,700	56,724,719	15,597,711,769	97.05
RU1R	51,622,244	15,486,673,200	50,096,084	13,745,318,767	97.04
RU2B	55,437,688	16,631,306,400	53,829,737	14,808,721,643	97.1
RU2R	57,702,160	17,310,648,000	55,876,299	15,366,298,591	96.84
RU3B	61,207,662	18,362,298,600	59,271,682	16,298,065,800	96.84
RU3R	57,541,010	17,262,303,000	55,739,002	15,301,750,449	96.87

Table S3: Mutation information of common gene mutation sites

GroupID	ID	start	end	pos	target_AA	others_AA
Cluster1019	RU_DN287266_c0_g1_i1-D6	1	235	144	A	V
Cluster1020	RU_DN287266_c0_g1_i1-D8	1	206	135	N	S
Cluster1147	RU_DN27598_c0_g1_i4	66	1,106	389	P	T
Cluster1147	RU_DN27598_c0_g1_i4	66	1,106	1,086	T	N
Cluster1151	RU_DN716_c3_g1_i1	318	872	586	D	E
Cluster1151	RU_DN716_c3_g1_i1	318	872	637	S	N
Cluster1151	RU_DN716_c3_g1_i1	318	872	660	K	E
Cluster1151	RU_DN716_c3_g1_i1	318	872	756	A	T
Cluster1151	RU_DN716_c3_g1_i1	318	872	776	M	T
Cluster1193	RU_DN301746_c0_g1_i1-D13	1	60	26	E	D
Cluster1197	RU_DN78575_c0_g1_i3	11	340	90	R	H
Cluster1198	RU_DN97972_c0_g1_i4	333	711	530	P	S
Cluster1287	RU_DN23112_c1_g1_i1-D11	1	206	133	I	V
Cluster1312	RU_DN10511_c0_g1_i4	1	72	27	Q	H
Cluster1333	RU_DN7639_c1_g1_i1-D16	1	263	101	V	A
Cluster1404	RU_DN1145_c3_g2_i23-D404	1	87	14	A	V
Cluster1428	RU_DN11179_c0_g1_i1-D18	1	131	42	F	L
Cluster1437	RU_DN11179_c0_g1_i1-D26	1	113	69	P	S
Cluster1455	RU_DN2964_c0_g3_i1	1	1,008	749	S	N
Cluster797	RU.gth.013323.1-D19	1	107	85	A	P
Cluster877	RU_DN2388_c0_g1_i14-D2	8	77	21	A	E
Cluster896	RU_DN870_c0_g1_i2	22	113	42	F	Y

Cluster896	RU_DN870_c0_g1_i2	22	113	68	N	D
Cluster897	RU_DN870_c0_g1_i2-D13	1	272	179	R	K
Cluster928	RU_DN39010_c0_g1_i6	80	325	202	F	L
Cluster928	RU_DN39010_c0_g1_i6	80	325	307	V	L
Cluster941	RU_DN144928_c0_g1_i1-D15	19	87	25	H	Y
Cluster958	RU_DN30740_c0_g1_i1-D39	1	293	46	Q	H
Cluster980	RU_DN104765_c0_g1_i1-D111	1	275	226	S	L
